# Supplementary material for: Highly Mesoporous g-C3N4 with Uniform Pore Size Distribution via the Template-Free Method to Enhanced Solar-Driven Tetracycline Degradation
Source: Nanomaterials (Basel). 2021 Aug 11;11(8):2041. doi: 10.3390/nano11082041 (PMC8398958; doi:10.3390/nano11082041)
Supplement: Supplementary file 1 [file nanomaterials-11-02041-s001.zip › nanomaterials-1181641-supplementary.pdf]

Supplementary data

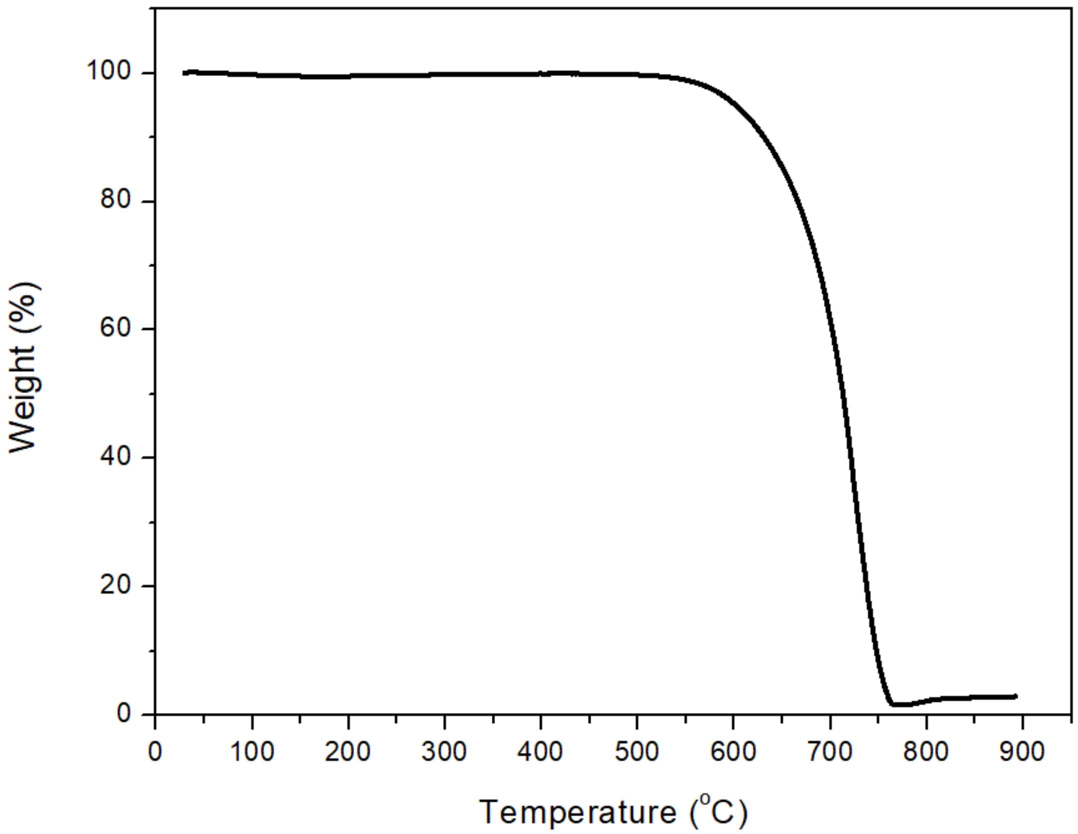

Figure S1. The TGA of GCN-B.

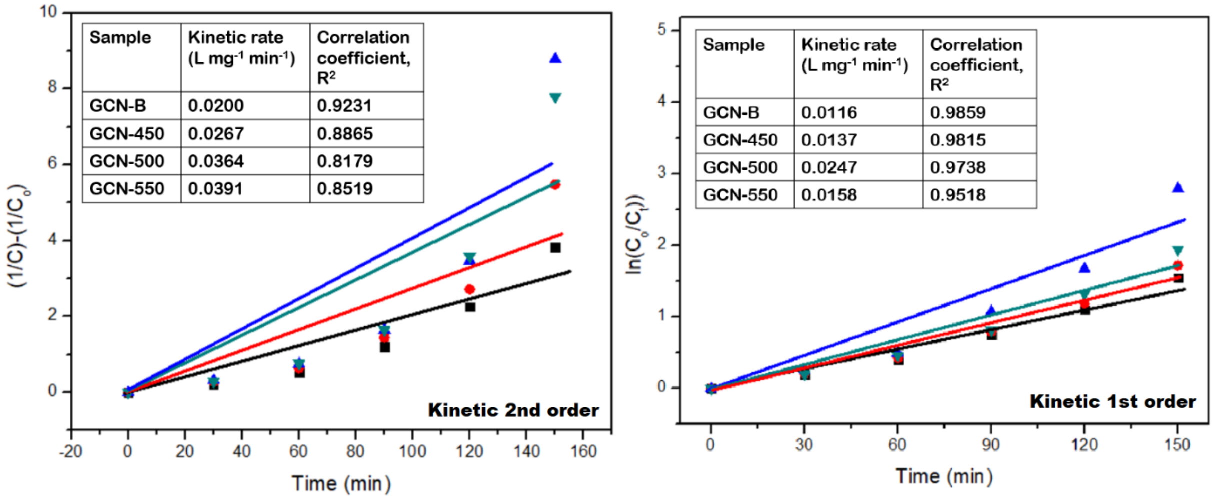

Figure. S2. Fitting curve of GCNs using different kinetics reaction equation.

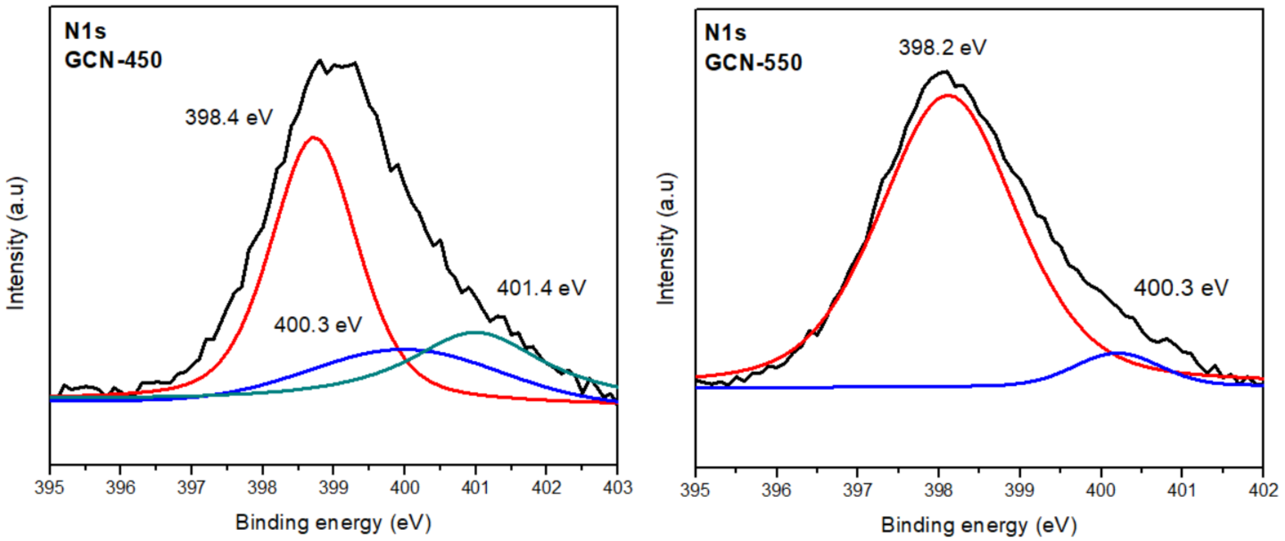

Figure. S3. The XPS of GCN-450 and GCN-550.

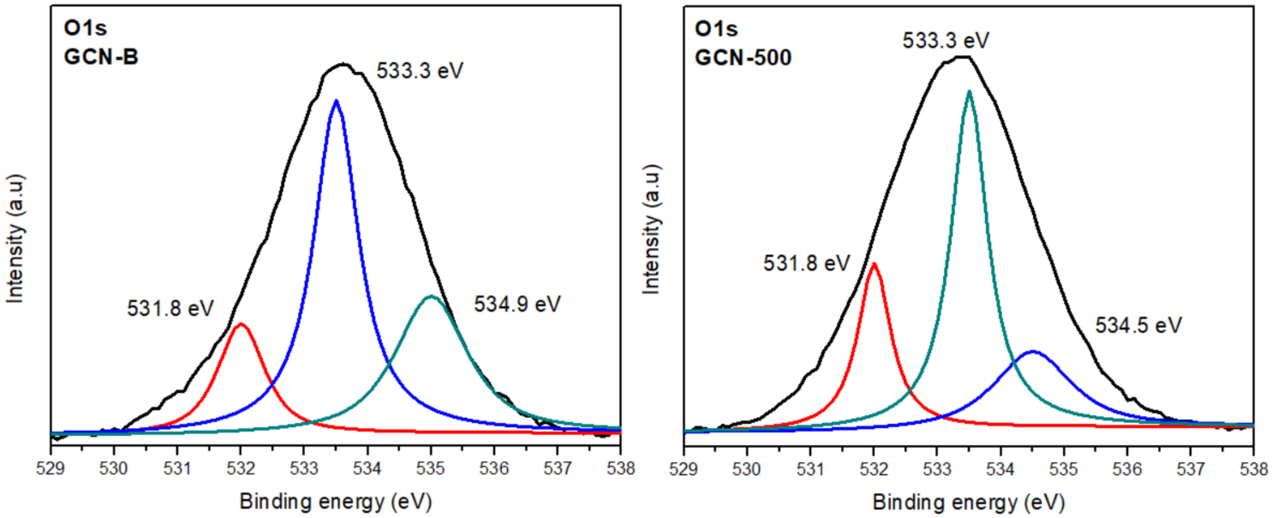

Figure. S4. The O1s peak of GCN-B and GCN-500.
